# Supplementary material for: Lipidomic and biophysical homeostasis of mammalian membranes counteracts dietary lipid perturbations to maintain cellular fitness
Source: Nat Commun. 2020 Mar 12;11:1339. doi: 10.1038/s41467-020-15203-1 (PMC7067841; doi:10.1038/s41467-020-15203-1)
Supplement: Supplementary file 5 — Description of Additional Supplementary Files [file 41467_2020_15203_MOESM5_ESM.pdf]

Title: Supplementary Data
Description: Raw lipidomes. Lipid species abundances (in picomoles) for all samples reported in this manuscript.
